# Impact of oxidative stress on female reproductive parameters: an analysis of systemic and follicular biomarkers

**DOI:** 10.5935/1518-0557.20250047

**Published:** 2025

**Authors:** Aline Q. Rodrigues, Guilherme G. Carvalho, Tathyana B. Piau, Fabiane H. Veiga, Paulo E. N. Souza, Daniel C. Moreira, Natalia I. Z. Tierno, Yamara A. Macedo, Maria Eduarda B. Amaral, Hitomi M. Nakagawa, Jair T. Goulart, Fernanda Paulini

**Affiliations:** 1 University of Brasilia, Institute of Biological Sciences, Department of Physiological Sciences, Brasilia-DF, 70910-900, Brazil; 2 University of Brasilia, Faculty of Health Sciences and Technologies, Ceilandia campus, Brasilia-DF, 70910-900, Brazil; 3 University of Brasilia, Institute of Physics, Laboratory of Electron Paramagnetic Resonance, Brasilia-DF, 70910-900, Brazil; 4 University of Brasilia, Faculty of Medicine, Morphology and Applied Immunology Research Center, Brasília - DF, 70910-900, Brazil; 5 Maternal and Child Hospital of Brasilia Dr. Antonio Lisboa, Assisted Reproduction Sector, Brasilia-DF, 70203-900, Brazil; 6 Genesis - Human Reproduction Assistance Center, Brasilia-DF, 70390-700, Brazil

**Keywords:** female fertility, assisted reproduction, redox balance, oxidative biomarkers, reproductive parameters

## Abstract

**Objective:**

To examine the relationship between oxidative stress markers and reproductive parameters in women undergoing assisted reproductive treatments, focusing on the interaction between demographic, oxidative, and reproductive factors.

**Methods:**

A total of 49 patients with a mean age of 36.0±3.5years were evaluated. Biomarkers analyzed included reduced glutathione (GSH), disulfide glutathione (GSSG), total glutathione (tGSH), the GSSG/GSH ratio, reactive oxygen species (ROS), and reactive nitrogen species (RNS). Blood and follicular fluid samples were collected and analyzed, correlating these markers with variables such as antral follicle count (AFC), oocytes retrieved, mature oocytes, blastocyst formation, and pregnancy rates.

**Results:**

The results showed that AFC is a key marker of ovarian reserve, correlating positively with the number of oocytes retrieved and mature oocytes. Age significantly influenced antioxidant markers in both blood and follicular fluid, with a decline in antioxidant capacity linked to aging. In follicular fluid, higher GSSG levels correlated with the number of oocytes retrieved, indicating increased metabolic activity and ROS generation, thus raising oxidative burden during ovarian stimulation. Additionally, GSH and tGSH levels in blood negatively correlated with mature oocytes, suggesting greater antioxidant consumption during elevated metabolic demand.

**Conclusions:**

Glutathione is a robust and clinically promising redox biomarker for female fertility, evidencing strong correlations between systemic and follicular redox markers. Advancing age and repeated ovarian stimulation were shown to compromise antioxidant capacity, negatively impacting reproductive outcomes. Establishing reference values, particularly for the GSH/GSSG ratio, may enable its use as a prognostic tool, while the quantification of ROS and RNS could support personalized interventions and ovarian stimulation protocols.

## INTRODUCTION

Fertility is a significant concern for global public health due to its intrinsic connection to reproductive health and the overall well-being of both men and women. The World Health Organization defines infertility as a disease and estimates that one in six individuals worldwide experiences difficulties conceiving (World Health Organization, 2023). Assisted Reproductive Technologies (ART), such as in vitro fertilization (IVF) by conventional insemination and intracytoplasmic sperm injection (ICSI), provide effective alternatives for addressing infertility (Njagi *et al*., 2020; Brautsch *et al*., 2023).

In recent years, scientific advances in ART have been remarkable, driven by an intensified search for biomarkers capable of evaluating oocyte quality and predicting pregnancy outcomes (Alfaidy *et al*., 2019; Pizarro *et al*., 2020). Procedures such as conventional IVF and ICSI facilitate the precise selection of gametes and the identification of embryos with the highest potential for successful implantation. Nevertheless, oocyte quality remains a limiting factor in achieving successful treatment outcomes, as cells with suboptimal characteristics are directly linked to the development of less viable embryos, thereby compromising reproductive success (Alfaidy *et al*., 2019; Pizarro *et al*., 2020).

Compromised oocyte quality is closely associated with failures in development and maturation, with clinical pregnancy rates following follicular aspiration and embryo transfer for IVF reported to be 26.3% and 33.5%, respectively (Smeenk *et al*., 2024). In this context, oocyte competence, defined as the ability to complete meiosis, undergo fertilization, and develop into a viable embryo, is a critical determinant of reproductive success. This process is highly complex and arises from a dynamic and coordinated interaction between the oocyte and its follicular microenvironment (Eppig *et al*., 1996; Da Broi *et al*., 2018; Malo *et al*., 2024). The health of the intrafollicular environment, sustained by cumulus cells and follicular fluid, is essential for oocyte maturation, as it provides vital metabolic and antioxidant support (Karabulut *et al*., 2020; Pizarro *et al*., 2020; Schütz & Batalha, 2024).

The follicular fluid, produced by granulosa cells during the later stages of follicular development, contains a diverse array of components, including steroid hormones, metabolites, proteins, polysaccharides, reactive oxygen species (ROS), and antioxidants. These elements play a pivotal role in supporting the growth and maturation of oocytes and follicular cells while protecting them from oxidative stress and damage (Da Broi *et al*., 2018; Zhang et al., 2020; Barroso-Villa *et al*., 2023). Alterations in the composition of follicular fluid, driven by hormonal, paracrine, and autocrine signaling pathways or systemic conditions, can adversely affect oocyte quality, thereby compromising fertilization success and embryonic development (Da Broi *et al*., 2018; Zhang *et al*., 2020; Barroso-Villa *et al*., 2023).

Among the components present in follicular fluid, ROS plays a significant role in oocyte quality. An imbalance between the production of these molecules and their neutralization by antioxidant systems leads to oxidative stress, which is a primary factor compromising oocyte quality (Karabulut *et al*., 2020). Deficiencies in antioxidant defense systems disrupt the cellular redox state, adversely affecting various female reproductive functions, including oocyte maturation and quality, corpus luteum formation, ovarian steroidogenesis, fertilization, embryonic development, and pregnancy success (Olszak-Wąsik *et al*., 2019; Karabulut *et al*., 2020).

Although oxidative stress is widely recognized as a key factor in the pathophysiology of infertility, its underlying causes, effects, and molecular mechanisms are still not fully understood (Olszak-Wąsik *et al*., 2019; Karabulut *et al*., 2020). Elevated levels of ROS, increased lipid peroxidation, and reduced antioxidant capacity in follicular fluid are likely associated with poor embryo quality and lower fertilization rates, however consensus on this correlation has yet to be reached (Mauchart *et al*., 2023). Regardless, it is well-established that the imbalance between ROS production and control by antioxidants causes damage to cellular components, leading to the loss of function of lipids, nucleic acids, carbohydrates, and proteins (Aitken *et al*., 2022; Mauchart *et al*., 2023).

Therefore, the identification and clinical validation of novel biomarkers capable of predicting oocyte competence to develop into a viable embryo with high implantation potential represents an innovative opportunity to improve the success rates. This advancement could facilitate the development of more effective and personalized treatments tailored to diverse patient profiles (ESHRE Guideline Group on the Number of Embryos to Transfer, 2024). In light of these considerations, the aim of this study was to explore the correlations between follicular and systemic oxidative stress in women undergoing infertility treatments and to evaluate their impact on oocyte quality, embryonic development, and pregnancy outcomes.

## MATERIAL AND METHODS

The study was approved in advance by the Human Research Ethics Committee of the Faculty of Health Sciences at the University of Brasília (CEP/UnB) and the National Research Ethics Commission (CONEP), under CAAE No. 4.882.986. All participants provided written informed consent by signing the Free and Informed Consent Form (FICF), ensuring their full understanding of the research objectives and procedures and their voluntary agreement to participate. The processes of the study are in accordance with the ethical standards defined by the committee responsible for studies involving humans and with the Declaration of Helsinki of 1964.

**Figure 1:**
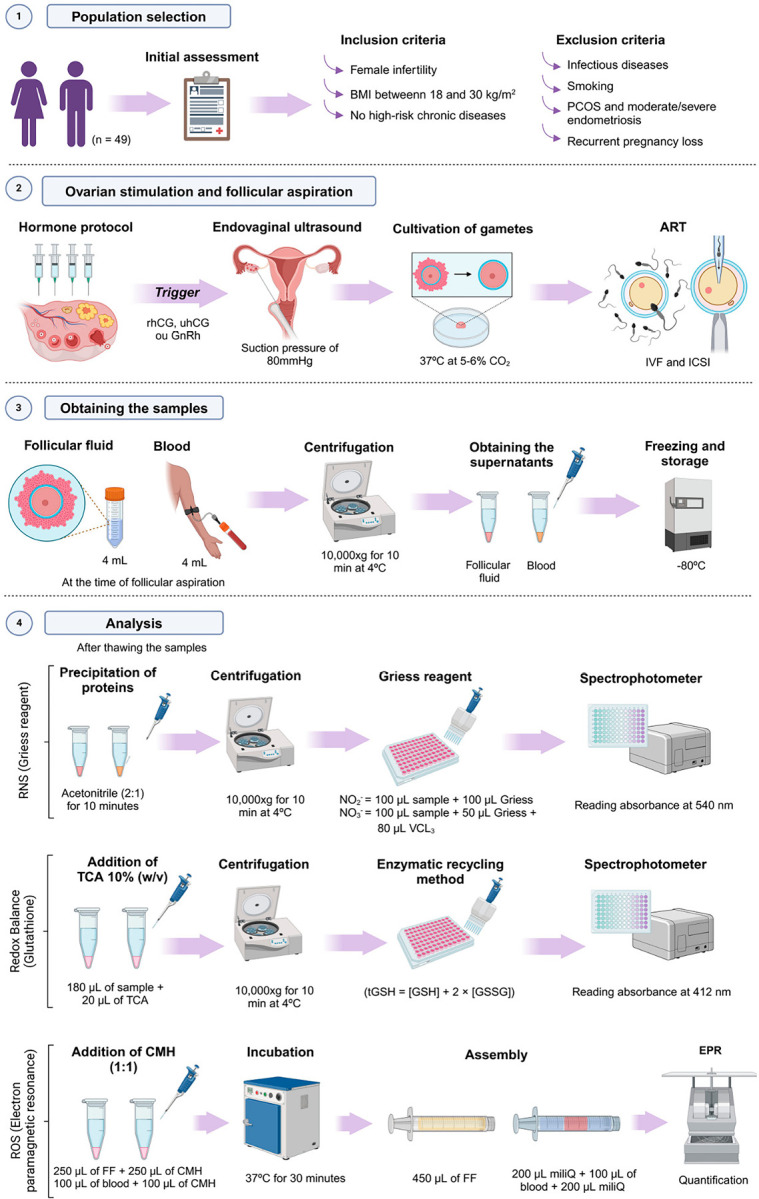
Experimental Design. Women diagnosed with infertility, with a body mass index (BMI) between 18 and 30 kg/m^2^ and without high-risk chronic diseases, were included in the study, while patients with infectious diseases, smoking, polycystic ovary syndrome (PCOS), moderate/severe endometriosis, or a history of recurrent pregnancy loss were excluded. Ovarian stimulation followed standard protocols, with medication adjustments based on individual characteristics. Follicular development was monitored via transvaginal ultrasound, and aspiration was performed after triggering with recombinant human chorionic gonadotropin (rhCG), urinary human chorionic gonadotropin (uhCG), and/or gonadotropin-releasing hormone (GnRH) agonist, once follicles reached ≥18mM in diameter. Retrieved gametes were cultured in specific media for conventional in vitro fertilization (IVF) or intracytoplasmic sperm injection (ICSI) and maintained in incubators at 37°C with 5-6% CO₂. During follicular aspiration, 4 mL of follicular fluid (FF) was collected from dominant follicles, with visibly contaminated samples discarded, and 4 mL of venous blood was simultaneously collected using tubes with heparin or EDTA. Samples were centrifuged at 10,000×g for 10 minutes at 4°C to separate plasma and remove cellular debris, with supernatants aliquoted into 500µL portions and stored at -80°C. Reactive nitrogen species (RNS) were quantified by treating samples with acetonitrile (2:1) for protein precipitation, followed by the addition of Griess reagent and absorbance reading at 540 nm. Glutathione levels were determined using the spectrophotometric enzymatic recycling method in samples treated with 10% trichloroacetic acid (TCA). Reduced glutathione (GSH), disulfide glutathione (GSSG), and the GSSG/tGSH ratio were measured. Reactive oxygen species (ROS) were quantified using electron paramagnetic resonance (EPR) after treating samples with CMH (carboxymethoxyhydroxyamine), incubating them at 37°C for 30 minutes before freezing and storage. ART=assisted reproductive technologies; VCl3=vanadium chloride.

Figure 1 provides a detailed illustration of the experimental design adopted in this study, encompassing the inclusion and exclusion criteria for population selection as well as the laboratory procedures implemented for each analysis.

### Population selection

The study included 49 infertile patients (n=49) undergoing assisted reproduction treatment at the Centro de Assistência em Reprodução Humana - Genesis and the Hospital Materno Infantil de Brasília (HMIB), both located in Brasília, DF, Brazil. Inclusion criteria were established to ensure homogeneity within the study group and comprised a prior diagnosis of female infertility, a body mass index (BMI) between 18 and 30 kg/m^2^, and the absence of high-risk chronic diseases that could compromise a healthy pregnancy. Exclusion criteria included positive serology for infectious diseases (HIV, HTLV, syphilis, hepatitis B, and hepatitis C), smoking, diagnoses of polycystic ovary syndrome (PCOS) or moderate to severe endometriosis, as well as a history of recurrent pregnancy loss.

### Ovarian stimulation and follicular aspiration protocols

The follicular aspiration protocols implemented at the Centro de Assistência em Reprodução Humana - Genesis and the Hospital Materno Infantil de Brasília (HMIB) are designed based on individualized medical criteria. These protocols aim to address the specific needs of each patient and optimize the quality of oocytes retrieved for assisted fertilization procedures.

Ovarian stimulation was performed using medications adjusted according to the patient’s age, antral follicle count, availability of medical supplies, and the clinical judgment of the attending physician. The drugs utilized included menotropins (Menopur^®^ and Merional^®^), urofollitropin (Fostimon^®^), and recombinant FSH (Gonal^®^). Stimulation continued until the follicles reached the target diameter, typically 18mM or greater, as determined by ultrasound evaluation.

In general, final follicular maturation, commonly referred to as the trigger, was achieved when ultrasound identified at least two follicles with a diameter of ≥18mM. Triggering was induced through the administration of recombinant human chorionic gonadotropin (rhCG), urinary-derived human chorionic gonadotropin (uhCG), or a gonadotropin-releasing hormone (GnRH) agonist. Follicular aspiration was performed 35 to 36 hours post-trigger to retrieve the oocytes. The ovarian stimulation protocols were personalized for each patient. As a result, some patients received the GnRH antagonist protocol, while others were treated with progesterone, based on their individual needs and clinical judgment. This personalized approach allowed for better management of the ovarian response and ensured the proper timing of ovulation for each patient.

For follicular aspiration, patients arrived at the clinical centers fasting, in accordance with established clinical guidelines. The procedure was performed using 17-gauge single-lumen needles (CooperSurgical^®^ Wallace^®^ Single Lumen Oocyte Recovery System, USA) under transvaginal ultrasound guidance (Logic GE^®^ P5). Prior to the collection, the needles were washed with a heparinized culture medium (phosphate-buffered saline; Ingamed, Maringá, Brazil) supplemented with 25 IU/mL heparin (Cristália, São Paulo, Brazil). The culture medium was maintained at a temperature between 25°C and 37°C, with a pH of 7.3±0.1 and osmolarity adjusted to 280-288 mOsm/L.

During follicular aspiration, needles were connected either to an aspiration pump (Pioneer Pro-Pump^®^ Dual - Cooper Surgical) with a constant negative pressure of 80mMHg to 110mMHg or, at HMIB, to syringes manually operated by the clinician. In the laboratory, follicular fluid was analyzed under stereoscopic microscopy to identify the cumulus-oophorus complex. The oocytes were subsequently transferred to plates containing specialized culture media for gametes and embryos and maintained in incubators with strict environmental controls, including 5-6% CO₂, a temperature of 37°C, and a stabilized pH of 7.29. Only mature oocytes (Metaphase II) were selected for subsequent procedures such as vitrification, IVF or ICSI. It is important to note that while a small number of patients in the study initially opted for oocyte vitrification as part of fertility preservation, these patients have not yet utilized their vitrified oocytes. Therefore, the data included in this study pertains solely to the analysis of fresh embryo transfers. All patients underwent IVF/ICSI with fresh oocytes, and no PGT-A was performed during the study period.

Fertilization was evaluated approximately 17 hours after microinjection, and embryo culture was maintained for up to six days under the same optimized conditions. In both clinics, biochemical pregnancy detection was performed using a human chorionic gonadotropin (Beta-hCG) test, with values exceeding the predefined laboratory reference threshold (>25 mIU/mL). This test was conducted 12 to 14 days after embryo transfer in accordance with established clinical protocols. Clinical pregnancy was confirmed by the visualization of a gestational sac via ultrasound, providing definitive evidence of embryo implantation.

### Obtaining Follicular Fluid and Blood

During the follicular aspiration procedure, approximately 4 mL of follicular fluid were collected from each patient, extracted from one or more dominant follicles (≥18mM). The samples underwent an initial visual inspection and were discarded immediately if contaminated with blood. The follicular fluid was then centrifuged at 10,000×g for 10 minutes at 4°C to remove cellular debris. Following centrifugation, the samples were divided into 500µL aliquots and prepared for the specific analyses described below. Simultaneously, 4 mL of blood were drawn using heparinized vacuum collection tubes or tubes containing ethylenediaminetetraacetic acid (EDTA), with a 24-gauge needle. Blood collection was performed at the same time as follicular aspiration, prior to the patient being placed under anesthesia. The blood samples were centrifuged at 10,000×g for 10 minutes at room temperature to separate the plasma. The supernatant was then collected in 500µL aliquots and prepared for subsequent analytical procedures.

### Analysis of Reactive Nitrogen Species (RNS)

To initiate the analysis, the samples were removed from the ultrafreezer and samples were thawed under on ice. Acetonitrile was added to the aliquots in a 2:1 ratio to precipitate proteins. The samples were placed on ice for 10 minutes to ensure protein precipitation. Following this, they were centrifuged at 10,000×g for 10 minutes at 4°C to separate the protein and liquid fractions. The resulting supernatants were carefully collected for nitrate (NO₃⁻) and nitrite (NO₂⁻) quantification. The strong relationship between the production of nitric oxide (NO) and the concentrations of nitrite and nitrate (NOx) in biological fluids like plasma, serum, and urine makes these markers valuable for accurately quantifying NO levels in vivo (Granger *et al*., 1996).

For NO₂⁻ determination, 100µL of the supernatant was mixed with 100µL of Griess reagent containing 0.1% (w/v) N-(1-naphthyl) ethylenediamine (NED) and 2% (w/v) sulfanilamide (SULF). This process was conducted in triplicate in sterile 96-well plates, and the samples were incubated at room temperature for 10 minutes, following the method described by Giustarini *et al*. (2008).

For NOx quantification, 100µL of the supernatant was combined with 80µL of vanadium chloride (VCl₃) and 50µL of Griess reagent containing 0.1% (w/v) NED and 2% (w/v) SULF. Incubation VCl₃ reduces all NO₃⁻ to NO₂⁻, which then reacts with Griess reagents (Miranda *et al*., 2001). These samples were also prepared in triplicate using sterile 96-well plates and incubated at 37°C for 30 minutes. Standard curves for both assays were generated using serial dilutions of NO₃⁻ and NO₂⁻ at concentrations of 200, 100, 50, 12.5, 6.25, and 3.125µM. Absorbance was measured at 540 nm using a microplate reader, enabling quantification of reactive nitrogen species (RNS) in the samples. The levels of NO₃⁻ were calculated as the difference between NOx and NO₂⁻ concentrations.

### Analysis of Redox Balance

Following sample collection, 180µL of follicular fluid or blood plasma were carefully transferred and mixed with 20µL of trichloroacetic acid (TCA) 100% (w/v) to achieve a final concentration of 10% TCA, stabilize samples and precipitate proteins. The stabilized samples were promptly immersed in liquid nitrogen at -196°C for rapid freezing and subsequently stored at -80°C until further analyses were conducted. On the day of measurement, samples were thawed under on ice before centrifugation at 10,000xg for 10 minutes at 4°C.

The concentrations of GSSG and total glutathione (tGSH=[GSH] + 2 × [GSSG]) were quantified using an enzymatic recycling method (Griffith, 1980) adapted for microplate analysis (Rahman *et al*., 2006). This method is based on the ability of thiol groups (-SH) to reduce the disulfide bond of DTNB (5,5’-dithiobis-2-nitrobenzoic acid), resulting in the formation of TNB (2-nitro-5-thiobenzoate), a compound that absorbs light at 412 nm.

In a reaction medium containing glutathione (GSH and/or GSSG), DTNB, glutathione reductase (GR), and NADPH, the rate of absorbance change at 412 nm (∆A412/min) is directly proportional to the total concentration of glutathione.

To determine tGSH levels, GSH standard solutions were prepared at concentrations of 5, 10, 20, 40, and 80 μM. A standard curve was generated by applying 10μL of each standard solution to a final reaction volume of 200μL. The reaction medium consisted of 125mM phosphate buffer (pH 7.0), 1.25mM EDTA, 0.5% (w/v) TCA, 0.2mM NADPH, 0.27mM DTNB, and 0.27 U/mL GR. For the sample analysis, the standard solution was replaced with the acid supernatant.

To specifically quantify GSSG, samples and GSSG standard solutions were incubated with 20mM 2-vinylpyridine for 1h to derivatize GSH before setting the assay in a 96-well microplate (Griffith, 1980). Standard solutions of GSSG were at concentrations of 0.2, 0.4, 0.8, 1.6, and 3.2 μM. A standard curve was generated by applying 20μL of each standard solution to a final reaction volume of 200μL. The reaction medium consisted of 125mM phosphate buffer (pH 7.0), 1.25mM EDTA, 0.2mM NADPH, 0.4mM DTNB, and 0.4 U/mL GR.

Each standard curve point and sample were analyzed in triplicate using a 96-well microplate. The reaction was initiated by adding NADPH, and absorbance at 412 nm was monitored for two minutes. Both the standard curve and the samples were measured simultaneously on the same plate. The rates of absorbance change for the samples were compared with the standard curve, and the concentrations of total glutathione (tGSH) and disulfide glutathione (GSSG) were calculated. The concentration of reduced glutathione (GSH) was determined by subtracting the GSSG concentration from tGSH. The GSSG/GSH ratio was calculated by dividing the respective concentrations.

### Analysis of Reactive Oxygen Species (ROS)

For the quantitative analysis of ROS, the electron paramagnetic resonance (EPR) technique was employed. Upon sample collection, 250µL of follicular fluid was immediately treated with a working solution containing 400mM CMH (carboxymethoxyhydroxyamine), 25mM DF (desferroxamine mesylate), 5mM DETC (diethyldithiocarbamate), and 100 U/mL heparin sodium in a 1:1 ratio. The mixture was incubated at 37°C under gentle agitation for 30 minutes. Subsequently, 450µL of the solution was transferred to a 1 mL container and immediately immersed in liquid nitrogen at -196°C. The samples were stored at -80°C until analysis. All solutions were prepared in Krebs-HEPES buffer (KHB, Noxygen, Elzach, Germany), with the pH adjusted to 7.4 using deionized double-distilled water, immediately prior to the experiments.

For the blood analyses, 100µL was immediately treated with a working solution containing 400mM CMH (carboxymethoxyhydroxyamine), 25mM DF (desferroxamine mesylate), 5mM DETC (diethyldithiocarbamate), and 100 U/mL sodium heparin in a 1:1 ratio. The tube containing the mixture was incubated at 37°C under gentle agitation for exactly 30 minutes. Following incubation, 100µL of the solution was placed in an uncapped 1 mL container and positioned between two ice blocks containing 200µL of Milli-Q water each. The samples were then immediately immersed in liquid nitrogen at -196°C and subsequently stored in a freezer at -80°C until further analysis.

### Statistical Analysis

A descriptive analysis of the variables was initially conducted, including the calculation of means and standard deviations. The normality of the data was then evaluated using the Shapiro-Wilk test to determine the most suitable correlation method. Spearman correlation analyses were performed to explore linear associations between continuous variables such as age, BMI, antral follicle count (AFC), number of oocytes retrieved, mature oocytes, and levels of reactive oxygen species (ROS) and reactive nitrogen species (RNS) in blood and follicular fluid. The correlation coefficients (r) were interpreted in terms of the strength and direction of the associations, while the corresponding p-values were calculated to assess statistical significance. All statistical analyses were performed using GraphPad Prism 9.0 software (GraphPad, San Diego, USA). A *p*-value<0.05 was considered statistically significant for all analyses.

## RESULTS

### Clinical Characteristics and Demographic Profile of the Patients

**Figure 2:**
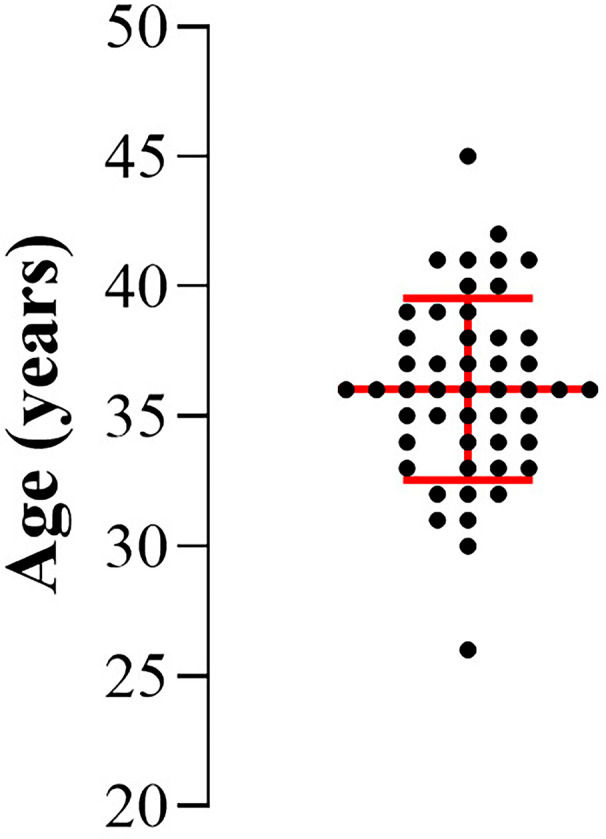
Age distribution of the patients assessed in the study, represented by individual points. The central red line indicates the mean and the upper and lower horizontal lines represent the standard deviations, showing the homogeneity of the group in relation to age.

The study included 49 patients whose profiles were assessed based on descriptive variables essential for understanding reproductive outcomes. Among the analyzed data, age emerged as a significant factor, displaying excellent normality and homogeneity, with a mean of 36.0±3.5years. This finding underscores the consistency of the study group concerning this parameter. The age distribution of patients is depicted in Figure 2.

Table 1 presents the primary clinical parameters analyzed in the study, including the minimum, maximum, mean, and standard deviation values. The variables encompass patients’ age, BMI, antral follicle count (AFC), duration of infertility, number of oocytes retrieved, mature oocytes, and blastocysts. The data presented below provide valuable insights into the patients’ profiles and reproductive outcomes.

**Table 1:** Clinical parameters assessed in the study, including age, body mass index (BMI), antral follicle count (AFC), duration of infertility, number of oocytes retrieved, mature oocytes, and blastocysts, presented with their minimum and maximum values, means, and standard deviations (±SD).

Clinical parameters			
	**Minimum**	**Maximum**	**Mean±SD**
Age (years)	26	45	36.0±3.5
BMI (kg/m^2^)	22.5	29.7	24.6±2.4
AFC (n)	4	49	15.7±9.4
Duration of infertility (years)	1	24	6.4±3.8
Number of oocytes retrieved	2	31	11.2±6.6
Number of mature oocytes	0	26	8.2±5.6
Number of blastocysts	0	12	2.3±2.8

The mean age of 36years, combined with the wide variability in antral follicle count (AFC), underscores the inclusion of patients of advanced reproductive age, where a decline in ovarian reserve is anticipated. However, the heterogeneous responses to ovarian stimulation highlight individual variability. The average number of oocytes retrieved (11.2±6.6) and mature oocytes (8.2±5.6) indicates a generally positive response among many patients, though the range (minimum of 2 retrieved and 0 mature) reveals significant challenges for some, likely linked to diminished oocyte reserve or quality. The average of 2.3±2.8 blastocysts formed per patient, with a range from 0 to 12, reflects variability in embryonic development capacity, potentially influenced by factors such as age, duration of infertility, and gamete quality. Additionally, the average BMI of 24.6±2.4 falls within the healthy range, though the proximity to overweight (maximum of 29.7 kg/m^2^) in some patients warrants consideration as a potential factor affecting reproductive outcomes.

**Figure 3:**
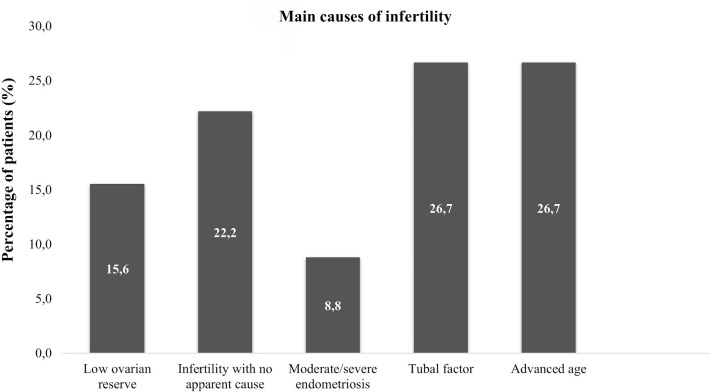
Percentage distribution of the main causes of female infertility identified in the study.

The average duration of infertility observed in the study was 6.3years, underscoring the prolonged and challenging journey these women endure, from their initial attempts to conceive to ultimately seeking specialized medical assistance. This study examined the primary causes of infertility that led patients to pursue assisted reproduction. Figure 3 illustrates the percentage distribution of these causes, highlighting the most prevalent factors.

The data presented in Figure 3 highlight that tubal factors and advanced age are the primary causes of female infertility, followed by unexplained infertility and low ovarian reserve. Low ovarian reserve is more commonly associated with reduced success rates in IVF/ICSI cycles rather than natural pregnancy rates. In this study, low ovarian reserve was considered a cause of infertility specifically within the context of assisted reproduction. The impact of low ovarian reserve on natural pregnancy rates was not the focus of the study and is not addressed here. Additionally, the high prevalence of unexplained infertility underscores the diagnostic challenges and multifactorial nature of infertility, emphasizing the need for tailored, patient-specific approaches.

Table 2 complements these findings by presenting the distribution of the purposes for which treatments were conducted. Furthermore, the analysis of reproductive outcomes, including both positive and negative results, provides a more comprehensive perspective on the effectiveness of the procedures. This view highlights the diverse motivations for seeking assisted reproduction and offers valuable insights into the success rates and challenges associated with the treatments. It is important to note that clinical pregnancy rates were determined exclusively for patients who underwent conventional IVF or ICSI.

**Table 2:** Distribution of the purposes of the treatments conducted and the clinical pregnancy outcomes among the patients evaluated in the study. The purposes include *in vitro* fertilization (IVF), intracytoplasmic sperm injection (ICSI), and fertility preservation. Outcomes are presented as positive and negative clinical pregnancies, expressed in absolute numbers and percentages, highlighting the predominant therapeutic choices and their corresponding success rates.

Purpose of processing				Pregnancy	
	**IVF**	**ICSI**	**Fertility preservation**	**Positive**	**Negative**
Number of patients	1	34	14	15	13
Percentage (%)	2.0	69.4	28.6	53.6	46.4

**Figure 4:**
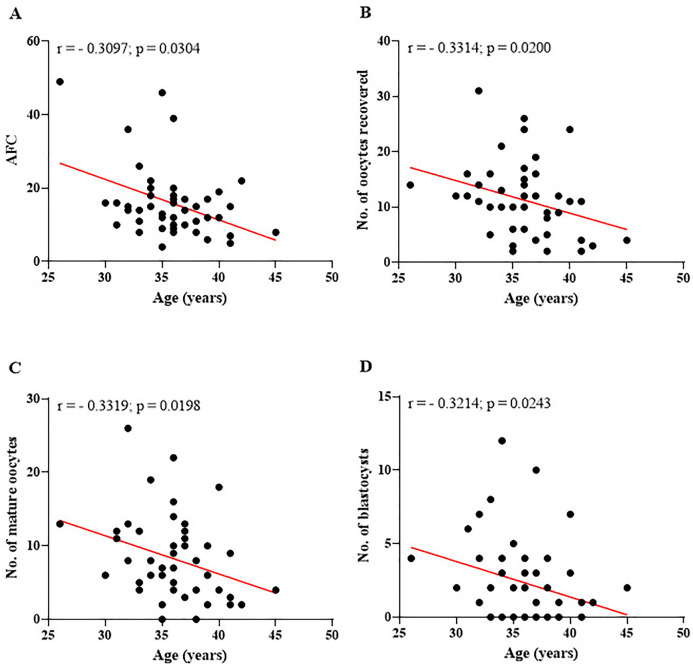
Relationship between patient age and key reproductive parameters assessed. (A) Antral follicle count (AFC) significantly decreases with advancing age. (B) A negative correlation is observed between the number of oocytes retrieved during ovarian puncture and age. (C) A negative correlation is also evident between the number of mature oocytes and age. (D) The number of blastocysts formed shows a significant decline with increasing age. r=Spearman’s correlation coefficient; No.=number. Significant differences are noted when - *p*-value<0.05.

This personalized approach becomes even more significant when considering the data presented in Table 2, which identifies ICSI as the most frequently utilized procedure, accounting for 69.4% of cases. In light of this, several clinical indicators were analyzed collectively to provide a deeper understanding of how demographic characteristics influence reproductive parameters. Figure 4 illustrates the correlations between participants’ age and the reproductive indicators evaluated, emphasizing the effects of aging on ovarian reserve, oocyte quality, and the capacity to form viable embryos. This analysis highlights the critical role of age in determining reproductive outcomes.

**Figure 5:**
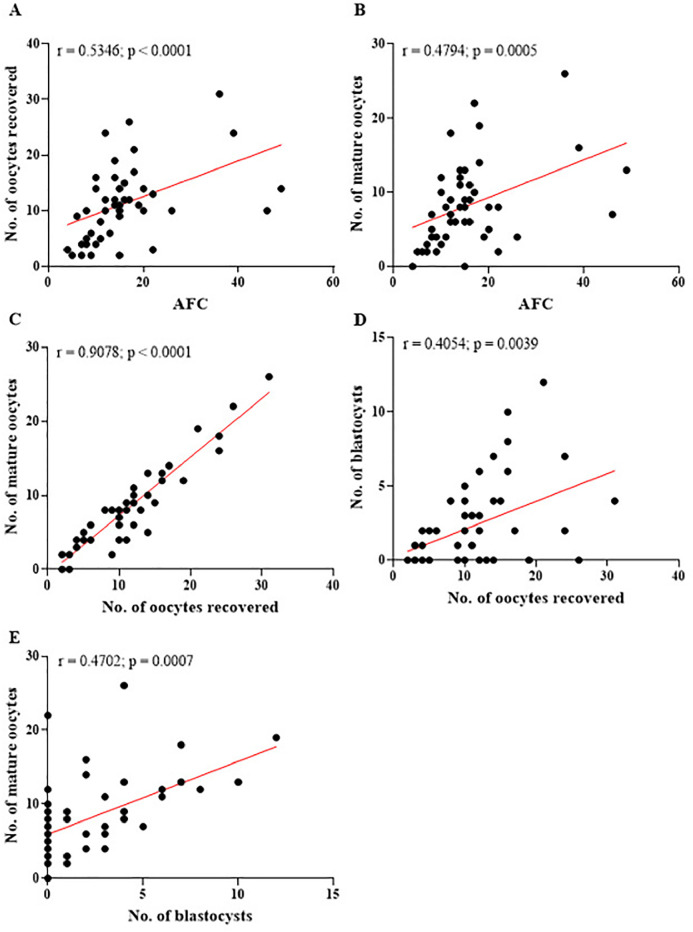
Correlations between the reproductive parameters evaluated. (A) Positive correlation between antral follicle count (AFC) and the number of oocytes retrieved. (B) Positive correlation between AFC and the number of mature oocytes. (C) Positive correlation between the number of oocytes retrieved and the number of mature oocytes. (D-E) Positive correlation between the number of oocytes retrieved and mature oocytes with the number of blastocysts formed. r=Spearman’s correlation coefficient; No.=number. Significant differences are noted when *p*-value<0.05.

The results presented in Figure 4 clearly demonstrate the negative impact of age on all the reproductive parameters assessed. As age increases, there is a progressive decline in AFC (Figure 4A), indicating a reduction in ovarian reserve, a critical factor for reproductive capacity. This decline is also evident in the number of oocytes retrieved during follicular aspiration (Figure 4B), showing that fewer follicles are punctured as age advances. As shown in Figure 4C, older women have fewer oocytes that reach maturity, which compromises the subsequent stages of assisted reproduction. Furthermore, Figure 4D illustrates that aging directly impacts blastocyst formation, a crucial stage for embryo implantation and achieving a successful pregnancy. Complementing these analyses, Figure 5 displays the correlations between key reproductive parameters, highlighting positive and significant relationships between AFC and oocyte parameters. The graphs also emphasize the interaction between the number of oocytes retrieved and those reaching maturity, as well as the influence of these factors on blastocyst formation.

Figure 5 (A-B) demonstrates a significant positive correlation between AFC and the number of oocytes retrieved, as well as between AFC and the number of oocytes that reached maturity during assisted reproduction treatments. These results reinforce that women with a higher antral follicle count have better reproductive prospects, given their greater availability of viable and mature oocytes for fertilization. This perspective is further supported by the data presented in Figure 5 (C), which demonstrates a strong positive correlation (*r*=0.9078; *p*<0.0001) between the number of oocytes retrieved and those that reached maturity. This indicates that patients with a higher number of punctured oocytes tend to achieve better results in terms of oocyte maturation. This finding is closely linked to ovarian reserve, measured by AFC, which directly affects the number of oocytes available for fertilization. Additionally, Figure 5 (D) highlights a significant, albeit more moderate, positive correlation (*r*=0.4054; *p*=0.0039) between the number of oocytes retrieved and the number of blastocys

### Oxidative parameters: biomarkers and reproductive impacts

#### Oxidative stress markers: ROS and RNS

The analysis revealed that the average concentration of reactive oxygen species (ROS) in the follicular fluid was 52.20±40.08µM, while in blood, this concentration was significantly lower, at 28.40±11.57µM. However, a high degree of variability was observed in the samples. In follicular fluid, ROS concentrations ranged from 11.63µM to 187.2µM, while in blood, concentrations ranged from 8.01µM to 68.16µM. The patient with the lowest concentration of ROS in follicular fluid (11.63µM) was 31years old, BMI of 19 kg/m^2^, AFC of 10, and retrieved 16 oocytes, of which 12 were mature, resulting in 6 blastocysts. She underwent ICSI and achieved a successful pregnancy despite having unexplained infertility, which had persisted for only 1 year. Conversely, the patient with the highest concentration of ROS in follicular fluid (187.2µM) was 38years old, had a BMI of 26.6 kg/m^2^, an AFC of 8, and retrieved 5 oocytes, of which 4 were mature, resulting in 2 blastocysts. Despite her infertility being caused by low ovarian reserve and lasting for 4years, she also achieved a positive pregnancy outcome (data not shown). This highlights that while lower ROS concentrations may correlate with better outcomes, successful pregnancies can still occur with elevated ROS, underlining the physiological role of ROS and the importance of individualized clinical approaches and tailored treatments in reproductive success. Table 3 below summarizes the oxidative parameters measured in the blood and follicular fluid, highlighting the minimum, maximum and mean values±standard deviations (±SD).

**Table 3:** Oxidative parameters evaluated in blood and follicular fluid, including minimum, maximum, mean (± SD) values for reactive oxygen species (ROS), reactive nitrogen species (RNS), reduced glutathione (GSH), disulfide glutathione (GSSG), GSSG/GSH ratio (%), and total glutathione (tGSH).

Oxidative parameters						
	**Blood**			**Follicular fluid**		
	**Minimum**	**Maximum**	**Mean±SD**	**Minimum**	**Maximum**	**Mean±SD**
ROS(µM)	8.01	68.16	28.4±11.57	11.63	187.20	52.2±40.8
RNS (µM)	23.48	267.17	61.0±39.39	12.09	122.84	47.91±21.23
GSH (µM)	0.14	16.8	3.45±2.98	0.33	11.81	3.75±2.9
GSSG (µM)	0.02	11.78	1.27±2.23	0.04	3.06	1.07±0.69
GSSG/GSH (%)	2.14	73.7	21.23±14.8	3.49	64.29	27.6±15.43
tGSH (µM)	0.44	25.5	4.72±4.94	0.65	14.45	4.82±3.25

For reactive nitrogen species (RNS), assessed as total NOx (sum of NO₃⁻ and NO₂⁻ concentrations), the averages were 47.91±21.23µM in follicular fluid and 61.0±39.39µM in blood plasma. Similarly to ROS, RNS also exhibited highly variable minimum and maximum levels. In follicular fluid, concentrations ranged from 12.09µM to 122.84µM, while in blood plasma, they varied widely from 23.48µM to 267.17µM. This variability may stem from factors similar to those influencing ROS levels.

**Figure 6:**
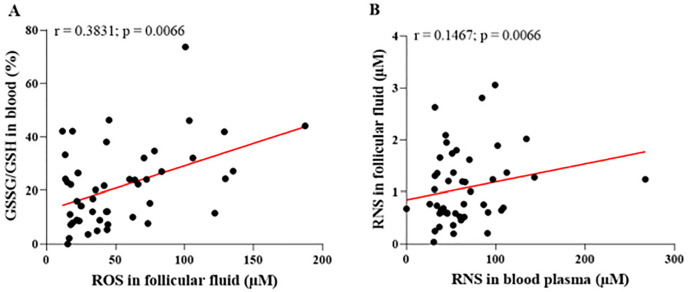
Correlations between oxidative stress markers in blood and follicular fluid. (A) A significant positive correlation between concentrations of reactive oxygen species (ROS) in follicular fluid and the GSSG/GSH ratio in blood, indicating that increased systemic oxidative stress may directly influence the follicular microenvironment. (B) A significant positive correlation between reactive nitrogen species (RNS) levels in blood plasma and follicular fluid, suggesting a link between oxidative states in the two compartments. GSSG=disulfide glutathione; GSH=reduced glutathione; r=Spearman’s correlation coefficient. Significant differences are noted when *p*-value<0.05.

A statistically significant correlation was observed between RNS concentrations in the blood and the follicular microenvironment, indicating distinct dynamics in the distribution or production of these reactive species. Figure 6 illustrates the correlations between oxidative stress markers, specifically examining the relationship between ROS in follicular fluid and the GSSG/GSH ratio in blood, as well as between RNS concentrations in blood plasma and follicular fluid.

The positive correlation between ROS concentrations in follicular fluid and the GSSG/GSH ratio in blood, as shown in Figure 6 (A), suggests that systemic oxidative stress is directly linked to its intensification within the follicular environment. However, the data in Figure 6 (A) reveal that as ROS levels in follicular fluid increase, the GSSG/GSH ratio in blood also rises, indicating a more oxidized systemic redox state. Figure 6 (B) illustrates a significant positive correlation between RNS levels in blood plasma and follicular fluid, suggesting functional integration between systemic and local oxidative stress compartments.

#### Redox balance

**Figure 7:**
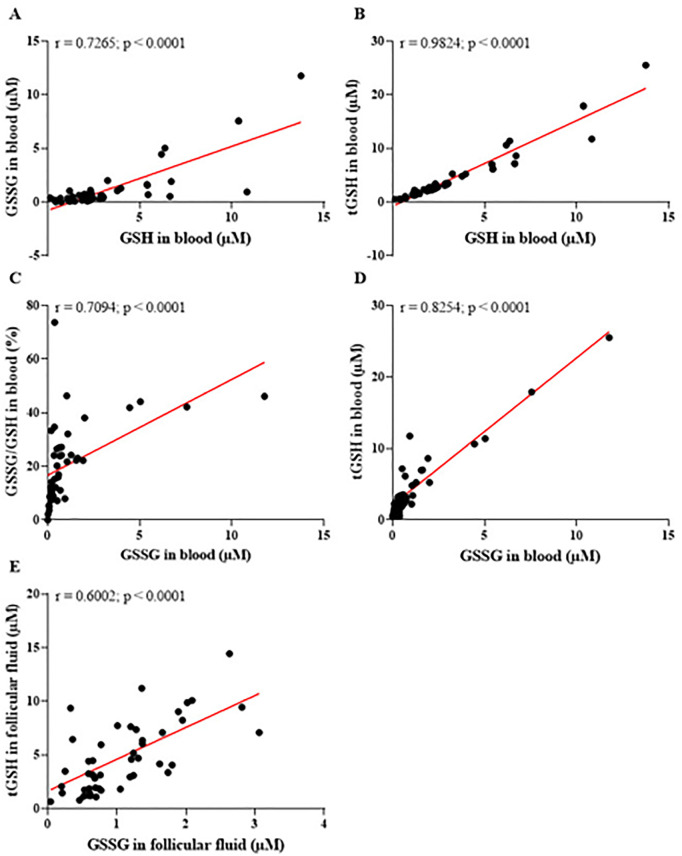
Correlations between different glutathione markers in blood and follicular fluid, assessing systemic and local antioxidant capacity. (A) A significant positive correlation between disulfide glutathione (GSSG) and reduced glutathione (GSH) levels in blood, reflecting a functional balance of the redox system. (B) A positive correlation between total glutathione (tGSH) and GSH in blood, highlighting the predominant role of GSH in contributing to the total antioxidant capacity. (C) A significant positive correlation between the GSSG/GSH ratio and GSSG levels in blood, suggesting that an increased oxidative load shifts the redox balance toward a more oxidized state. (D) A significant positive correlation between GSH and GSSG in blood, illustrating an adaptive antioxidant response to oxidative stress. (E) A significant positive correlation between GSH and GSSG in follicular fluid, indicating that the follicular microenvironment mirrors the systemic redox state and endeavors to preserve its local antioxidant equilibrium. r=Spearman’s correlation coefficient. Significant differences are noted when *p*-value<0.05.

The redox balance markers evaluated in this study are directly linked to glutathione, a key endogenous antioxidant critical for buffering oxidative stress in the human body. The mean concentration of GSH in the follicular fluid was 3.75±2.89µM, while GSSG levels were 1.07±0.69µM, with a GSSG/GSH ratio of 27.57±15.59%. In blood plasma, the mean concentrations of GSH were 3.44±3.0µM, GSSG 1.27±2.25µM, and the GSSG/GSH ratio was 21.23±14.91% (data not shown). Figure 7 illustrates the correlations between various glutathione markers in blood and follicular fluid, emphasizing the interconnected functioning of the antioxidant system across these compartments. This underscores the systemic and local roles of glutathione in modulating oxidative stress, particularly within the context of follicular development and reproductive health.

The positive correlation between GSSG and GSH levels in the blood (*r*=0.7265; *p*<0.0001), as shown in Figure 7 (A), suggests a dynamic equilibrium between the regeneration of GSH and its conversion to GSSG. This finding indicates that the patients’ antioxidant system is functional and actively responding to oxidative stress. However, the significant correlation observed between the GSSG/GSH ratio and GSSG levels (Figure 7C) highlights that, under conditions of heightened oxidative load, the redox system may shift towards a more oxidized state.

The results presented in Figure 7 (B) demonstrate a strong positive correlation between total glutathione (tGSH) and reduced glutathione (GSH) levels in the blood (*r*=0.9824; *p*<0.0001), highlighting that total antioxidant capacity is largely driven by GSH concentrations. This finding reinforces the pivotal role of GSH as the primary cellular antioxidant, crucial for neutralizing free radicals and protecting reproductive cells from oxidative damage.

Figure 7 (D) reveals that, despite the increase in GSSG levels, GSH also rose proportionally. This indicates that the patients’ antioxidant system is actively adapting to counter oxidative stress, as the elevation in GSH reflects the body’s capacity to maintain a total antioxidant reserve even under heightened oxidative load. However, this adaptation may also signal an overload of the redox system, where increased GSSG levels reflect a constant mobilization of antioxidant defenses to neutralize ROS.

In the follicular microenvironment, the data shows a significant positive correlation between GSH and GSSG (*r*=0.6002; *p*<0.0001) (Figure 7E), suggesting that the local antioxidant state partially mirrors the systemic state. This indicates that while there is a direct relationship between antioxidant markers in blood and follicular fluid, the local redox balance is not entirely dependent on the circulatory system.

**Figure 8:**
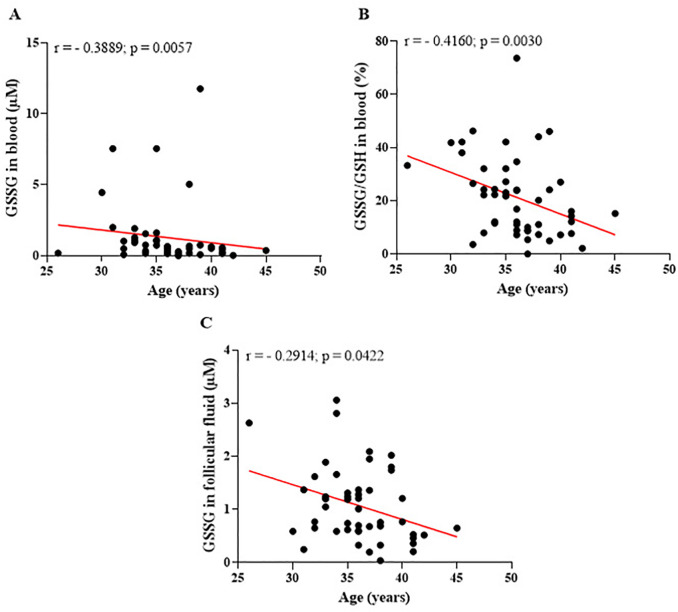
Relationship between patient age and glutathione markers in blood and follicular fluid. (A) Significant negative correlation between disulfide glutathione (GSSG) levels in blood and patient age, suggesting a diminished ability to recycle GSH into GSSG with increasing age. (B) Significant negative correlation between the GSSG/GSH ratio in blood and patient age, indicating a decline in the efficiency of systemic redox balance as women age. (C) Significant negative correlation between GSSG levels in follicular fluid and patient age, reflecting a reduction in local antioxidant capacity within the follicular microenvironment with advancing age. r=Spearman’s correlation coefficient. Significant differences are noted when *p*-value<0.05.

Regarding reproductive and demographic parameters, this study evaluated the relationship between the patients’ age and glutathione markers, offering an integrated perspective on the effects of aging on the antioxidant system. Figure 8 presents the results of these analyses, highlighting how age influences the balance of glutathione markers and their potential implications for reproductive health and outcomes.

**Figure 9:**
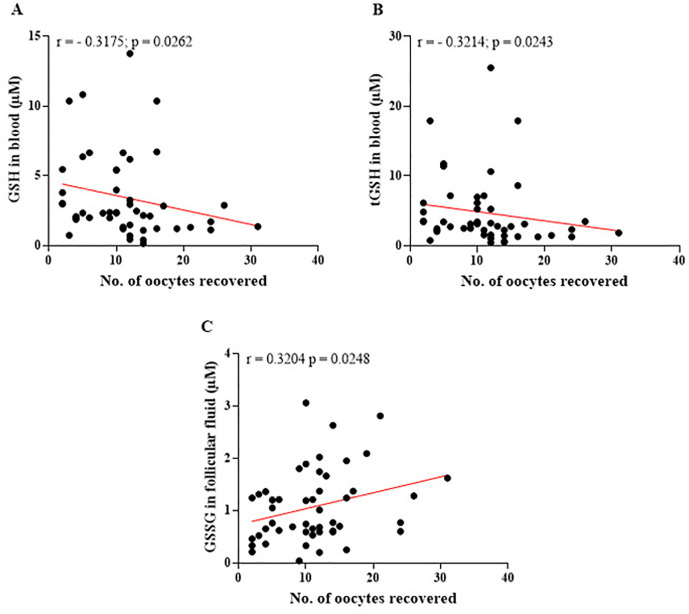
Relationships between the levels of glutathione markers in blood and follicular fluid and the number of oocytes retrieved. (A) A significant negative correlation between the levels of reduced glutathione (GSH) in blood and the number of oocytes retrieved, suggesting that an increase in oocyte retrieval is associated with higher consumption of systemic antioxidants. (B) A significant negative correlation between total glutathione (tGSH) levels in blood and the number of oocytes recovered, indicating the impact of ovarian stimulation on systemic antioxidant capacity. (C) A significant positive correlation between disulfide glutathione (GSSG) levels in follicular fluid and the number of oocytes retrieved, reflecting intensified local metabolic activity and an increase in the generation of reactive oxygen species (ROS) in the follicular environment with the increase in the number of oocytes obtained. *r*=Spearman’s correlation coefficient. Significant differences are noted when *p*-value<0.05.

The data presented in Figure 8 illustrate the impact of aging on the efficiency of the antioxidant system, both systemically and within the follicular microenvironment. In Figure 8 (A), the negative correlation (*r*=0.3889; *p*=0.0057) between GSSG levels in the blood and patient age suggests that, over time, GSSG levels decline. In addition, the relationship between the levels of glutathione markers in blood and follicular fluid and the number of oocytes recovered during reproductive treatments was analyzed. The findings reveal notable differences in the behavior of systemic and local antioxidants in relation to the number of gametes retrieved. These results underscore the critical role of redox balance in influencing ovarian response and gamete quality. Figure 9 illustrates these correlations, providing insights into how the oxidative state is modulated by the number of oocytes recovered during assisted reproduction treatments.

**Figure 10:**
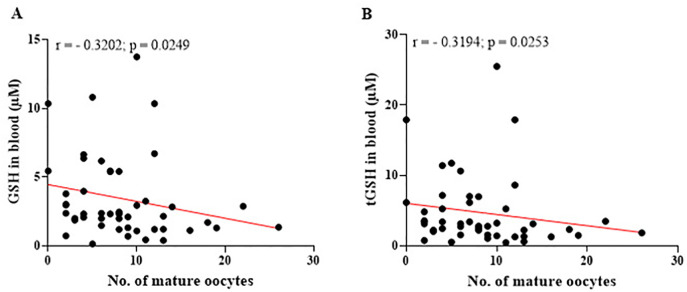
Relationship between the levels of reduced glutathione (GSH) and total glutathione (tGSH) in the blood and the number of mature oocytes. (A) Significant negative correlation between GSH levels in the blood and the number of mature oocytes, indicating increased consumption of systemic antioxidants during the metabolically demanding oocyte maturation process. (B) Significant negative correlation between tGSH levels in the blood and the number of mature oocytes, reflecting a reduction in overall antioxidant capacity due to heightened oxidative stress and metabolic activity. r=Spearman’s correlation coefficient. Significant differences are noted when *p*-value<0.05.

In the blood, the results suggest that as the number of recovered oocytes increases, there is a greater consumption of systemic antioxidants, likely due to the heightened metabolic demand and increased oxidative stress induced by ovarian hyperstimulation (Figure 9 A-B). Figure 10 illustrates the relationship between GSH and tGSH levels in the blood and the number of mature oocytes obtained during reproductive treatments. These graphs provide insights into how the consumption of systemic antioxidants correlates with the process of oocyte maturation, a metabolically demanding phase characterized by increased production of ROS. The data emphasize the dynamic interaction between systemic antioxidant capacity and the oxidative demands associated with oocyte maturation, highlighting the potential impact of redox balance on the quality and viability of mature oocytes during assisted reproduction treatments.

The graphs presented in Figure 10 (A-B) reveal a negative correlation between GSH and tGSH levels (*r*=- 0.3204; *p*=0.0249 and *r*=- 0.3194; *p*=0.0253, respectively) in the blood and the number of mature oocytes. These findings suggest that as the number of mature oocytes increases, levels of these biomarkers decrease, reflecting greater systemic antioxidant consumption to manage the oxidative stress generated during the oocyte maturation process.

## DISCUSSION

In assisted reproduction studies, where factors such as age play a central role, maintaining a homogeneous group is crucial to minimize bias. With a mean age of 36, the participants in this study represent women in a critical phase of their reproductive life, marked by challenges such as declining ovarian reserve and deteriorating oocyte quality. These changes, extensively documented in the literature, are linked to biological processes such as an increased risk of embryonic aneuploidies and reduced mitochondrial efficiency (du Fossé *et al.,* 2020; Ye *et al.*, 2023; Zhang & Wu, 2023).

According to the literature, populations within this age group face significant reproductive challenges yet retain a reasonable chance of success, as evidenced by the 59.8% pregnancy rate reported among patients undergoing conventional IVF and ICSI (Ovarian Stimulation TEGGO *et al*., 2021). However, while age homogeneity is a methodological strength, it also limits the generalizability of the results to women outside this age range. For instance, younger women (<35years) typically exhibit higher ovarian reserve (AFC) and better oocyte quality, whereas women of advanced reproductive age (>35years) experience more pronounced declines in both oocyte quantity and quality (Ye *et al.*, 2023). Therefore, although the findings are highly relevant to the typical assisted reproduction demographic, their extrapolation to extreme age groups should be approached with caution.

The study by Havrljenko *et al*. (2023) evaluated the predictive outcomes in 490 patients over the age of 35 undergoing an IVF cycle, emphasizing the detrimental effects of advanced maternal age on reproductive parameters. The researchers identified a significant decline in the average number of oocytes retrieved, metaphase II oocytes, and developed embryos as age increased. Furthermore, they highlighted that, for women in this age group, achieving an optimal live birth rate or cumulative live birth rate typically requires retrieving 10 - 12 oocytes in metaphase II or developing 10 - 11 embryos. These findings underscore the critical need for personalized strategies aimed at maximizing oocyte recovery in older women to enhance the success rates of assisted reproduction treatments.

Female infertility can arise from various causes, which not only guide the selection of treatment strategies but also significantly influence the potential outcomes (Xie *et al*., 2022; Zheng *et al*., 2023). In this study, tubal factors and advanced age are the main causes of female infertility. This distribution aligns with existing literature, which identifies advanced age as a key determinant in reduced fertility (du Fossé *et al*., 2020; Ye *et al.*, 2023; Zhang & Wu, 2023). Tubal factors, such as obstructions or damage to the fallopian tubes, often linked to previous infections or endometriosis, are also significant contributors to infertility and are commonly addressed through assisted reproduction techniques such as IVF (Yang *et al*., 2020a; Henry & Nisolle, 2024). Low ovarian reserve, another major factor, reflects a decline in the number of viable ovarian follicles, a condition exacerbated by age and sometimes genetic predisposition. Additionally, the high prevalence of unexplained infertility underscores the diagnostic challenges and multifactorial nature of infertility, emphasizing the need for tailored, patient-specific approaches.

In view of this, this study identified ICSI as the most frequently used procedure. The predominance of ICSI may be attributed to specific conditions, such as male infertility factors, low oocyte quality or the need to enhance the chances of success in IVF conventional, which necessitate advanced techniques to address challenges in fertilization effectively. Since only couples with female infertility were included in this study, it is likely that patients who underwent ICSI did so for reasons unrelated to male infertility. The technique is often employed as a control strategy in cases of inadequate fertilization during IVF cycles or as an effort to optimize outcomes for patients with a history of reproductive failure (Balli *et al*., 2022). In this context, the patients’ reproductive history, such as the number of cycles performed and previous outcomes achieved, represents a critical factor that was not analyzed but may directly influence the choice of technique.

Studies indicate that undergoing multiple cycles of assisted reproduction treatments can significantly increase physical and emotional stress, contributing to elevated levels of oxidative stress in patients. This imbalance negatively affects gamete quality, ultimately jeopardizing reproductive outcomes (Kushnir *et al*., 2022). The study by Negris *et al*. (2021) highlights the widespread perception among women undergoing fertility care that emotional stress adversely impacts reproductive outcomes. Among the 1,460 participants included in the study, 69.0% associated stress with reduced success in fertility treatments, and 31.3% felt it could lead to miscarriage. In response, the authors emphasized the need to address the psychological aspects of fertility care more comprehensively and informatively. Consequently, future studies incorporating variables such as reproductive history and the number of treatment cycles undertaken are crucial to deepening our understanding of these complex interactions. This highlights the importance of addressing not only biological but also emotional and psychological factors that influence fertility outcomes, especially ovarian reserve, thus influencing the quality and quantity of oocytes.

Several factors are recognized as predictors of ovarian response in assisted reproduction treatments, including age, baseline hormone levels (Follicle Stimulating Hormone, Luteinizing Hormone, estradiol, inhibin B, and Anti-Müllerian Hormone), and ultrasound measurements such as AFC. Recent studies, however, have identified AFC as the strongest predictor of ovarian reserve, outperforming AMH, BMI, and age. The European Society of Human Reproduction and Embryology recommends the use of AFC or AMH to predict high and low responses to ovarian stimulation, with a preference for AFC due to its superior accuracy and reliability in predicting reproductive outcomes (Lu *et al*., 2023; ESHRE Guideline Group on the Number of Embryos to Transfer *et al*., 2024; Dermolo *et al*., 2024). This finding in this study underscores the importance of AFC as a critical marker for predicting success in assisted reproduction cycles, serving as a valuable tool for planning and personalizing treatments.

The study by Dermolo *et al*. (2024), which evaluated 412 patients, demonstrated that those with an AFC of fewer than five follicles were 84% less likely to exhibit an ovarian response compared to patients with a higher AFC. The authors also identified a positive correlation between AFC and the number of oocytes retrieved, emphasizing its importance as a clinical predictor. Furthermore, their analyses confirmed that AFC was a more effective predictor of adverse outcomes than anti-Müllerian hormone (AMH), BMI or age. These findings align with the results of the present study, in which AFC exhibited a significant positive correlation with oocyte parameters, further reinforcing its critical role in predicting ovarian response.

In relation to ROS, it is known that these molecules are naturally produced during metabolism and regulated by antioxidant systems to maintain redox balance. This interplay forms a reactive species interactome (Cortese-Krott *et al*., 2017) that connects redox signaling to critical cellular functions, including protein regulation and stress responses (Sies & Jones, 2020). Disruptions in redox balance can lead to oxidative stress, characterized by molecular damage and impaired biomolecule function. Indeed, oxidative stress markers have been associated with several human diseases, including neurodegenerative, cardiorespiratory and reproductive diseases (Halliwell & Gutteridge, 2015). However, in many cases, the cause-and-effect relationship remains unclear. This is particularly true for infertility, where divergent results complicate understanding the role of changes in redox balance for pathophysiology.

This variability could be attributed to several factors. First, individual differences in oxidative stress levels may arise from variations in metabolic activity, underlying health conditions, or genetic predispositions that affect the production and neutralization of ROS (Kaltsas *et al*., 2023; Onaolapo *et al*., 2023; Zaha *et al*., 2023). Additionally, the degree of follicular development and ovarian stimulation protocols used in assisted reproduction treatments could influence ROS levels, as more metabolically active follicles may generate higher concentrations of ROS (Yang *et al*., 2020b; Mauchart *et al*., 2023; Henry & Nisolle, 2024). External factors such as diet, lifestyle, and environmental exposure to pro-oxidant agents could also contribute to the observed variability (de Angelis *et al*., 2020; Kaltsas *et al*., 2023). Furthermore, the variability in blood ROS concentrations might reflect systemic differences in the balance between oxidative stress and antioxidant defenses among patients, potentially influenced by overall health, inflammation, or oxidative conditions unrelated to reproduction. These findings highlight the complexity of oxidative stress dynamics and underscore the importance of considering individual variability in clinical and research settings.

In the follicular fluid, differences in RNS concentrations could be attributed to varying levels of local oxidative and nitrosative stress, influenced by the metabolic activity of individual follicles, the degree of inflammation in the ovarian microenvironment, or disparities in follicular development and ovarian stimulation protocols (Drejza *et al*., 2022; Awonuga *et al*., 2023; Barbosa *et al*., 2024). In blood plasma, the broader range of RNS concentrations likely reflects systemic factors, such as individual variations in inflammatory responses, overall metabolic health, or external influences like diet and environmental exposure. Genetic and hormonal factors regulating nitric oxide synthesis and degradation may also contribute to these discrepancies (Dutta & Sengupta, 2022; Treffon & Vierling, 2022).

The GSSG/GSH ratio serves as a sensitive indicator of the body’s redox state; physiologically, a low ratio reflects an efficient antioxidant system capable of neutralizing ROS and preserving cellular integrity (Alkazemi *et al*, 2021; Aitken *et al*., 2022). However, the observed elevated GSSG/GSH ratio indicates a heightened oxidative state, pointing to a condition in which antioxidant systems are unable to control reactive species at stress levels (Sies, 2021). This finding aligns with the arguments of Xiao & Loscalzo (2020), who highlight the GSH/GSSG system as the primary redox buffer within cells due to its high concentration relative to other redox pairs. Intracellularly, GSH plays a fundamental role in maintaining a highly reductive environment, with typical GSH/GSSG ratios in the cytosol ranging from ≥ 30:1 to 100:1.

Although an elevated GSSG/GSH ratio indicates a greater oxidative load, the positive correlation observed suggests that the patients’ antioxidant system is actively responding by adjusting the balance of GSSG and GSH in response to increased ROS. This reflects the body’s adaptive ability to manage oxidative stress and prevent oxidative damage from accumulating to some extent (Warzych & Lipinska, 2020). However, persistent reductions in GSH levels or a significant increase in the GSSG/GSH ratio can compromise this adaptive capacity, increasing susceptibility to oxidative stress. This, in turn, may negatively impact fundamental reproductive processes such as oocyte maturation, fertilization, and embryonic development (Karabulut *et al*., 2020; Zhang *et al*., 2020; Aitken *et al*., 2022; Barroso-Villa *et al*., 2023; Kaltsas *et al*., 2023). Research has shown that high GSH levels in follicular fluid are associated with improved fertilization rates and embryonic development. Conversely, an increased GSSG/GSH ratio is linked to reduced oocyte quality and impaired reproductive competence (Babayev & Duncan, 2022; Chen *et al*., 2023; Huang *et al*., 2023). Thus, while the observed positive correlation suggests an active antioxidant response, it also emphasizes the critical role of redox balance in supporting successful reproductive outcomes.

These findings imply that the follicular microenvironment is influenced by oxidative conditions in the bloodstream, supporting the hypothesis that systemic oxidative stress can directly affect the follicular environment (Mauchart *et al*., 2023; Zaha *et al*., 2023; Su et al., 2024). Previous studies reinforce this connection, demonstrating that elevated levels of free radicals are linked to DNA damage and dysfunction in granulosa cells, ultimately compromising oocyte viability and embryonic development (Mauchart *et al*., 2023; Zaha *et al*., 2023; Su *et al*., 2024).

These findings underscore the critical importance of controlling systemic oxidative stress to maintain a healthy follicular microenvironment and optimize reproductive outcomes. Strategies for monitoring and modulating oxidative status, including antioxidant supplementation or interventions designed to mitigate oxidative stress, should be explored as potential tools to enhance the success of assisted reproduction treatments. Such efforts are particularly relevant for vulnerable populations, such as women of advanced reproductive age or those undergoing multiple treatment cycles, where the risk of oxidative damage is heightened.

This study highlighted the variability in glutathione dynamics between systemic and local compartments, reflecting the body’s ability to adaptively manage oxidative stress in different environments (Babayev & Duncan, 2022; Chen *et al*., 2023; Huang *et al*., 2023). The higher GSSG/GSH ratio observed in the follicular fluid suggests a greater oxidative load in this microenvironment compared to blood plasma, emphasizing the importance of localized antioxidant defenses in preserving oocyte quality and supporting reproductive outcomes.

The study by Liu *et al*. (2021), which utilized infertile women as a control group, reported mean GSH concentrations of 1.57±0.35µM in blood plasma and 2.43±0.83µM in follicular fluid. These values are lower than those observed in our study. This discrepancy could reflect differences in the study populations, methodologies, or the severity of oxidative stress experienced by the patients. The relatively higher GSH levels observed in our study suggest a stronger antioxidant capacity in the patient cohort, potentially contributing to better reproductive outcomes. However, the variability in GSH concentrations underscores the need for individualized evaluation of oxidative stress markers to optimize treatment strategies in assisted reproduction.

In the context of reproduction, adequate GSH levels are particularly important, as they have been closely linked to key processes such as oocyte maturation and embryo viability (Aitken *et al*., 2022). This underscores the importance of maintaining optimal GSH concentrations to support a favorable redox balance, which is essential for ensuring successful reproductive outcomes in assisted reproduction treatments. Prolonged or intensified oxidative stress could eventually compromise these mechanisms, leading to insufficient protection against oxidative damage (Zhang *et al*., 2020; Aitken *et al*., 2022; Barroso-Villa *et al.,* 2023). In addition, elevated GSSG levels have been linked to negative effects on oocyte quality and the overall health of the follicular environment. The study by Babayev & Duncan (2022) reported that during ovarian stimulation cycles, there is a significant increase in GSSG levels within follicular fluid, indicative of heightened metabolic activity associated with local oxidative load. These findings highlight the dual nature of the redox system in responding to oxidative challenges: while it demonstrates resilience by increasing antioxidant reserves, excessive stress can still pose risks to reproductive outcomes by disrupting the delicate balance of the follicular microenvironment.

The significant correlation observed in this study between the GSSG/GSH ratio and GSSG levels highlights that, under conditions of heightened oxidative load, the redox system may shift towards a more oxidized state. This shift could potentially compromise the quality of the reproductive environment, as elevated oxidative stress may adversely affect oocyte quality and fertilization outcomes. These data align with findings from previous studies, which have demonstrated that an increased GSSG/GSH ratio is associated with reduced oocyte quality and impaired fertilization capacity (Zhang *et al*., 2020; Aitken *et al*., 2022; Barroso-Villa *et al*., 2023). Together, these results underscore the critical role of maintaining redox balance to ensure optimal conditions within the reproductive microenvironment, particularly in the context of assisted reproduction treatments.

The follicular microenvironment is influenced by additional factors, including the metabolism of granulosa cells, the permeability of the follicle-blood barrier, and the presence of specific hormones and nutrients (Onaolapo *et al*., 2023; Schütz & Batalha, 2024). These factors independently modulate the redox state, creating an antioxidant environment with unique characteristics. This finding is physiologically significant, as follicular fluid represents the immediate microenvironment surrounding oocytes. Its redox balance is crucial for protecting oocytes against oxidative damage, preserving their viability, and supporting subsequent stages of maturation and fertilization (Onaolapo *et al*., 2023).

The observed negative correlation between blood GSSG levels and patient age suggests that over time, GSSG levels decrease. This reduction may reflect a diminished ability of the body to recycle GSH into GSSG, signaling a weakening of the redox system’s efficiency. Such a decline could compromise total antioxidant capacity, which is crucial for protecting reproductive processes (Aitken *et al*., 2022; Barroso-Villa *et al*., 2023; Onaolapo *et al*., 2023). The GSSG/GSH ratio in blood and GSSG levels in follicular fluid also display negative correlations with age. These findings indicate that the redox balance becomes progressively less efficient as women age. This reduction in antioxidant capacity may result in a decreased ability to counteract oxidative stress that typically increases with aging. Consequently, these changes can adversely affect oocyte quality and embryo viability, emphasizing the critical role of maintaining a robust antioxidant defense system in preserving reproductive potential in older women.

To corroborate the results presented, the study by Seshadri *et al*. (2021) demonstrated that the follicular microenvironment of older women exhibits increased ROS levels along with a decline in antioxidant capacity, significantly impairing oocyte quality and the maturation process. This reduction in systemic antioxidant capacity highlights the significant impact of ovarian stimulation on the body’s redox balance. Conversely, in the follicular microenvironment, GSSG levels demonstrated a positive correlation with the number of oocytes retrieved. This proportional increase in local GSSG levels indicates that as more oocytes are obtained, metabolic activity in the follicular environment intensifies. Such elevated activity is typically associated with increased ROS production due to heightened cellular metabolism. While this is a normal physiological response during ovarian stimulation, excessive ROS levels can overwhelm the available antioxidant defenses in follicular fluid. This imbalance may compromise oocyte quality and reduce their fertilization potential, emphasizing the importance of maintaining a controlled oxidative environment during assisted reproduction treatments (Hamad *et al*., 2021; Aitken *et al*., 2022; Chen *et al*., 2023; Huang *et al*., 2023). Literature supports this observation, indicating that oocyte maturation is a metabolically intense process that significantly increases ROS production, thereby heightening the demand for systemic antioxidant mechanisms, such as glutathione (Zhang *et al*., 2020; Aitken *et al*., 2022).

Furthermore, the results highlight the influence of aging and ovarian stimulation on the antioxidant system, showing a reduced capacity to neutralize ROS in more vulnerable patients. These findings emphasize the importance of clinical strategies aimed at enhancing antioxidant protection for at-risk populations, such as older patients or those undergoing multiple cycles of ovarian stimulation. Future studies should aim to further investigate these mechanisms and their implications for reproductive outcomes, ultimately contributing to the optimization of assisted reproduction treatments.

## CONCLUSION

The results of this study highlight glutathione as a robust, sensitive, and clinically promising redox marker for the assessment of female fertility. Highly significant correlations were observed among the different glutathione markers in blood, particularly reduced glutathione (GSH), oxidized glutathione (GSSG), and total glutathione (tGSH), as well as between their levels in plasma and follicular fluid, suggesting a strong interdependence between systemic and local redox systems. This relationship strengthens the hypothesis that the peripheral redox profile may partially reflect the follicular microenvironment, offering a promising pathway for less invasive and more comprehensive clinical assessments.

Furthermore, the study demonstrated that advancing age and repeated cycles of ovarian stimulation progressively impair antioxidant capacity, negatively affecting reproductive parameters such as the number of mature oocytes, blastocyst formation, and pregnancy rates. The age homogeneity among participants contributed to more robust analyses, allowing the identification of consistent correlations between biomarkers and clinical indicators, such as the antral follicle count (AFC), thereby reinforcing the role of oxidative stress as a determining factor in oocyte q

Finally, for these biomarkers to be effectively applied in clinical practice, it is essential to establish reference values based on control groups composed of fertile women. The GSH/GSSG ratio, for example, shows potential as a prognostic tool for follicular quality. Based on these parameters, patients with unfavorable oxidative profiles could benefit from personalized interventions, such as antioxidant nutritional strategies. In addition, the quantification of ROS and RNS in blood samples may aid in the customization of ovarian stimulation protocols, contributing to improved clinical outcomes in assisted reproduction.

## Data Availability

The data supporting the findings of this article will be made available at reasonable request to the corresponding author.
